# Longitudinal changes in neural gain and its relationship to cognitive control trajectory in young adults with early psychosis

**DOI:** 10.1038/s41398-023-02381-x

**Published:** 2023-03-03

**Authors:** Bjorn Burgher, James Scott, Luca Cocchi, Michael Breakspear

**Affiliations:** 1 QIMR Berghofer Medical Research Institute, Brisbane, QLD Australia; 2 Child Health Research Centre, The University of Queensland, Brisbane, QLD Australia; 3 University of Newcastle, Newcastle, NSW Australia

**Keywords:** Schizophrenia, Neuroscience

## Abstract

The mixed cognitive outcomes in early psychosis (EP) have important implications for recovery. In this longitudinal study, we asked whether baseline differences in the cognitive control system (CCS) in EP participants would revert toward a normative trajectory seen in healthy controls (HC). Thirty EP and 30 HC undertook functional MRI at baseline using the multi-source interference task—a paradigm that selectively introduces stimulus conflict—and 19 in each group repeated the task at 12 months. Activation of the left superior parietal cortex normalized over time for the EP group, relative to HC, coincident with improvements in reaction time and social-occupational functioning. To examine these group and timepoint differences, we used dynamic causal modeling to infer changes in effective connectivity between regions underlying the MSIT task execution, namely visual, anterior insula, anterior cingulate, and superior parietal cortical regions. To resolve stimulus conflict, EP participants transitioned from an indirect to a direct neuromodulation of sensory input to the anterior insula over timepoints, though not as strongly as HC participants. Stronger direct nonlinear modulation of the anterior insula by the superior parietal cortex at follow-up was associated with improved task performance. Overall, normalization of the CCS through adoption of more direct processing of complex sensory input to the anterior insula, was observed in EP after 12 months of treatment. Such processing of complex sensory input reflects a computational principle called gain control, which appears to track changes in cognitive trajectory within the EP group.

## Introduction

Cognitive symptoms in early psychosis (EP) are a primary determinant of social and occupational outcomes [1]. Early intervention typically yields symptomatic and social-occupational recovery [2], but there are limited interventions to address cognitive impairment. Cognitive trajectories in EP vary from deteriorating to stabilizing or improving [3–7]. Understanding neural markers underlying cognitive trajectory may give therapeutic insights and endpoints to remediate functioning. Such an objective requires knowledge of cognitive and neurobiological changes over the course of psychotic disorders.

The examination of the cognitive trajectory in EP has often focused on executive function, due to its predictive value for clinical and social-occupational outcomes [8–10]. Cognitive control, a sub-dimension of executive function, appears sensitive to cognitive impairments. This is because cognitive control transcends traditional neuropsychological constructs in its role in maintaining external task focus required for many cognitive functions [11] by supporting neural dynamics within and across brain networks [12]. The cognitive control system (CCS) plays a hierarchical role in integrating sensory inputs and cognitive responses to support adaptive, goal-driven behavior [12–16]. This is important in complex and dynamic environments, such as employment and educational settings, where task execution depends on the ability to selectively focus on relevant information and suppress distractions.

This ability to integrate multimodal sensory input relies on a neural computation known as “gain”. Formally, the gain of a system corresponds to the gradient of its input–output ratio [17]. Gain hence supports cognition by selectively enhancing salient sensory inputs and suppressing distractors. Top-down gain control, by systems such as the CCS, corresponds to context-specific changes in this gradient. Dynamic causal modeling (DCM) is a generative modeling technique that parameterizes the gain between two regions using either a non-specified, additive input (“bilinear DCM”) or via hierarchical gating of coupled brain regions by the activity in a third region (“nonlinear DCM”) [18]. DCM has been used as a method of interrogating changes in effective connectivity in salience, central executive and reward networks in psychotic disorders [19–25], suggesting abnormal effective connectivity between frontal (dorsolateral prefrontal cortex (DLPFC), inferior and middle frontal gyrus, insula, anterior cingulate cortex (ACC)), temporal (hippocampus, middle temporal gyrus), parietal (inferior parietal sulcus, superior parietal cortex (SPC)), thalamic (medial dorsal thalamus), midbrain (ventral tegmental area and substantia nigra) and striatal (ventral striatum, pallidum) regions. The role of gain control in sensory processing in schizophrenia has recently been linked to the maintenance of cortical excitatory-inhibitory balance [26].

There are very few longitudinal functional neuroimaging studies examining the CCS in EP. A recent study examined the association between longitudinal changes in the CCS and behavioral performance on a continuous performance task [13]. This work suggested initial differences in both performance and functional connectivity within the CCS remained stable over 12 months [27]. However, studies relying on a phenomenological description like functional connectivity cannot characterize complex dependencies among cortical regions, since large-scale networks utilize hierarchical and causal interactions (i.e., effective connectivity). One study demonstrated restoration of hippocampal-frontal effective connectivity modulated by an episodic memory task in patients with schizophrenia after 1 week of antipsychotic treatment [28]. However, to-date there are no longitudinal studies evaluating task-modulated effective connectivity within executive function networks, such as the CCS, over an extended period to account for the effect of neurodevelopment. Characterizing changes in these dynamic neural exchanges longitudinally may capture how brain networks recover.

**Fig. 1:**
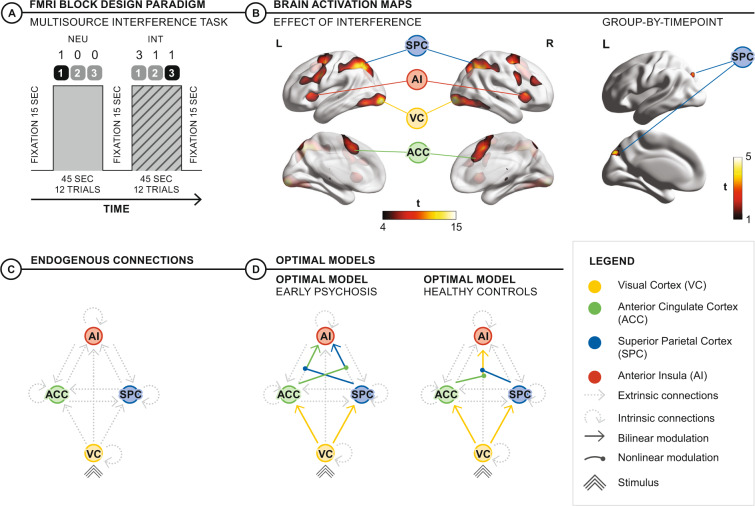
fMRI task and modeling. **A** Timing and example stimuli corresponding to neutral and interference conditions. Stimuli were presented for 2.5 s with individual trials organized in blocks of the same condition. Four blocks each of alternating neutral and interference conditions were interspersed by a fixation period. **B** Effect of Interference: the contrast of neutral <interference mapped to the cortical surface; canonical CCS regions showed strong and significant activation over timepoints (*p* < 0.05 FWE height threshold). The group-by-timepoint effect of interference was represented in a contiguous cluster extending the left-sided superior occipital cortex, precuneus and the superior parietal cortex (*p* < 0.001 uncorrected height threshold) **C** Endogenous connections: DCM model space common to all models (i.e., the A-matrix) where stimuli inputs via the VC. Stimuli then propagated via lower-order connections in a purely feedforward manner, with connections between ACC, SPC, and AI connected in a feedforward and feedback manner. **D** Different group optimal models at baseline: Although both groups favored nonlinear over bilinear models, the HC group used nonlinear modulations that directly gated stimulus conflict from the VC to AI in a single step. In contrast, the EP group employed an initial bilinear modulation from the VC to the ACC and SPC followed by parallel nonlinear gating on the subsequent connections to the AI.

The multi-source interference task (MSIT) is a well-validated task that selectively introduces stimulus conflict through spatial incongruence and visual distraction [29, 30]. The MSIT robustly activates the cingulo-opercular network—via its roles in sensory integration and target identification—plus the fronto-parietal network—via its role in context-specific behavioral control, attention and error feedback [12, 31]. Fronto-parietal network dynamics underlying the MSIT appear to be distinct from the traditional working memory n-back task, implying unique use of common neural substrates between cognitive control and working memory [32]. We recently reported slower reaction times in those with EP while resolving stimulus conflict in this task [25]. We then used DCM to infer how EP participants and healthy controls (HC) employ effective connectivity to resolve the stimulus conflict implicit in this task (Fig. 1D). HC participants employed a direct means of processing stimulus conflict, through nonlinear gating of input from the visual cortex (VC) to the anterior insula (AI) by the ACC and SPC, associated with faster responses. EP participants employed an indirect means of processing stimulus conflict, with initial linear modulations of input from the VC to the ACC and the SPC, before parallel nonlinear gating of inputs to the AI, associated with slower responses. We used computational methods to show how these differences in indirect versus direct nonlinear gating models mapped to group differences in gain control between stimulus inputs to the VC and task-related responses in the AI [33]. When transiting between neutral and interference conditions of the MSIT, the HC group employed a phasic change of high and low gain states between conditions whereas the EP group had tonic low gain across conditions. This difference in tonic versus phasic gain control demonstrates how the efficient integration of sensory-cognitive inputs by the CCS rests upon a flexible reorganization of neural processes as cognitive load increases.

Here, we report the follow-up of both groups at 12 months using fMRI and the MSIT, to observe how EP participants progressed when processing stimulus conflict after a period of assertive early intervention clinical care. We hypothesized that symptomatic and functional recovery would translate into improvements in task performance and a normalization of hierarchical cognitive control in EP participants. To achieve this, we employed Parametric Empiric Bayes (PEB) to study longitudinal changes in the bilinear and nonlinear DCMs from the baseline analysis of this cohort.

## Methods

### Participants

Thirty EP participants and thirty aged and gender-matched controls with no family history of mental health disorders were recruited from October 2017 to July 2019 for the baseline assessment. All participants were 17–25 years old. Participants were invited into a follow-up evaluation at 12 months of which 19 EP and 19 HC participants completed.

At follow-up, diagnosis (EP only) and substance use (both groups) were assessed using the Diagnostic Interview for Psychosis. The EP group were assessed using the Positive and Negative Symptom Scale (PANSS) [34] and Social and Occupational Functioning Assessment Scale (SOFAS) (Table 1) [35]. Medication type and antipsychotic dose were recorded (Table 1).

**Table 1:** Clinical and demographic information of participants.

	Healthy control Timepoint 1	Healthy control Timepoint 2	Early psychosis Timepoint 1	Early psychosis Timepoint 2
	*N* = 19		*N* = 19	
Characteristics	Mean (SD)		Mean (SD)	
Age (years)	21.8 (2.5)	22.8 (2.5)	21.2 (2.0)	22.2 (2.0)
Education (years)	15.3 (1.9)	15.7 (1.5)	14.1 (1.8)	14.9 (1.5)
WASI-II IQ estimate	115.5 (9.8)		109.2 (10.8)	
Chlorpromazine eq. (mg)			131.5 (114.1)	144.7 (123.8)
PANSS^a^			50.6 (21.2)	38.9 (7.9)
SOFAS^b^			55.7 (15.0)	68.3 (11.9)
	***N*** **(%)**		***N*** **(%)**	
Male	11 (57.8)		12 (63.1)	
Substance use^c^	6 (31.5)	6 (31.5)	11 (57.9)	10 (52.6)
Lithium			6 (31.5)	5 (26.3)
Combination			5 (26.3)	4 (21.1)
Schizophrenia			10 (52.6)	
Bipolar			9 (47.4)	

Participants were matched for age and gender. There were no significant differences between groups for education, IQ, and substance use.

^a^There was a significant decrease in PANSS from baseline to follow-up.

^b^There was a significant increase in SOFAS from baseline to follow-up.

^c^Substance use, defined by presence or absence of “high frequency” use of alcohol, tobacco, or illicit substance. High frequency was defined by scores greater than 3 (i.e., use of substance 3–4 times per week) on the diagnostic interview for psychosis.

All participants provided written informed consent and parental consent was also obtained for those under eighteen years of age. The study was approved by Royal Brisbane Women’s Hospital Human Research Ethics Committee (Ref. HREC/15/QRBW/613). All procedures in this work comply with the ethical standards of the relevant national and institutional committees on human experimentation and with the Helsinki Declaration of 1975, as revised in 2008.

### MRI data acquisition and pre-processing

Data at baseline and follow-up were acquired from a 3 T Siemens Prisma with a 64 channel radiofrequency head coil, including multiband (acceleration factor of 6) T2*-weighted echoplanar images (fMRI), spin echo field maps and T1-weighted MPRage images (sMRI). Pre-processing was completed using fmriprep v20.0.6 [36]. Framewise displacement was used to assess head motion of all participants.

### Task-related fMRI modeling

To investigate the effects of stimulus conflict on the CCS, fMRI data were acquired during the MSIT task (Fig. 1A) [29]. In brief, the MSIT presents stimuli organized into a group of three single digit numbers (0, 1, 2 or 3). One number is unique and the other two are the same as each other. During neutral blocks, the unique number is always in the spatial position congruent to the corresponding button and is flanked by zeros (e.g., ‘1 0 0’). These spatially congruent, distractor-free stimuli are associated with fast responses. During interference blocks, the unique number is not in its corresponding spatial position and is flanked by numbers (1,2,3) that are possible alternative responses (e.g., ‘2 1 2’). This requires participants to inhibit automatic stimulus-response congruent responses and select the appropriate button-press based on task-relevant information, with slower reaction time reflecting spatial incongruence and distraction effects.

A general linear model (GLM) of the fMRI data was performed in Statistical Parametric Mapping (SPM12, revision 7771) [37]. Trial blocks were convolved with a canonical hemodynamic response function and fitted to the blood oxygen level dependent (BOLD) time series at the subject level. T- and F-contrast images were created for the effect of stimulus conflict (interference > neutral trials) at both timepoints. Within subject t-contrasts were used to examine the effect of timepoint and brought to the second level to examine main effects (task, group, and timepoint) and their interaction. Statistical inference was performed with a cluster-level threshold of *p* < 0.05 (whole brain FWE corrected) following a default cluster-forming height threshold *p* < 0.001 (uncorrected). However, where effects are expressed very strongly, we report with a more stringent cluster-forming height threshold of *p* < 0.05 (FWE corrected)—this yields strong and relatively localized effects, consistent with the theoretical tenets of DCM [38].

**Fig. 2:**
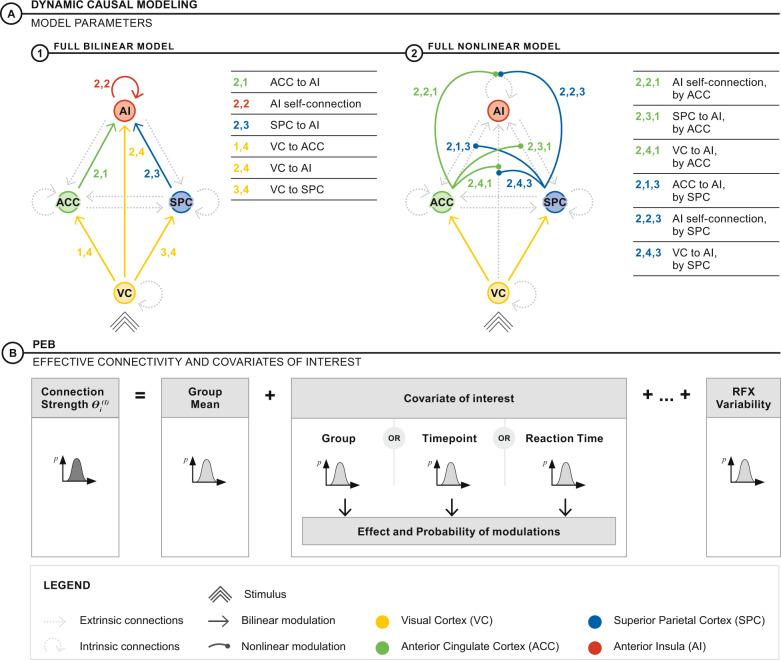
Dynamic causal modeling (DCM) and parametric empirical Bayes (PEB). **A** Two DCMs were used to explore the link between effective connectivity and other effects of interest (group, timepoint and reaction time). The full bilinear model (left) contains all possible additive, bilinear modulations on connections to the AI: Bold lines represent modulations turned “on” in the B-matrix. The full nonlinear model (right) contains all possible nonlinear modulations on connections to the AI, including the AI self-connection: Bold lines represent modulations turned “on” in the B- and D-matrix. **B** Subject level estimates of effective connectivity were extended to a hierarchical linear regression model exploring the effect of group status, timepoint, and reaction time in a random-effects analysis.

As previously reported, EP participants at baseline showed increased activation of the AI, modulated by the interference condition of the MSIT [28]. We used DCM [38] to provide plausible explanations of this effect through models of effective connectivity (directed neural interactions). As per our previous study, we used nonlinear DCM [39] to model how the ACC and SPC hierarchically gate input from the VC to the AI during trials of stimulus conflict [25]. This hierarchical gating, as represented by nonlinear effects, models neuromodulatory functions such as the short-term plasticity supported by NMDA receptors, that facilitate gain control at the level of cortical neurons [40]. Effective connectivity during neutral and interference trials were modeled with bilinear DCM (where coupled neural interactions are linearly up- or down-regulated by a task condition) and nonlinear DCM (where paired neural interactions are gated by a third brain region). The interference condition modulated all possible feedforward connections directed to the AI. Bilinear and nonlinear models mirror each other in terms of connections to the AI that could be modulated. However, nonlinear models prescribe that the gating modulation is explicitly enacted by a third brain region. Although both groups had distinctly different models of processing stimulus conflict in the previous study (Fig. 1D), this time we constructed a full bilinear model and a full nonlinear model that captured all possible modulations of the AI (Fig. 2A).

**Fig. 3:**
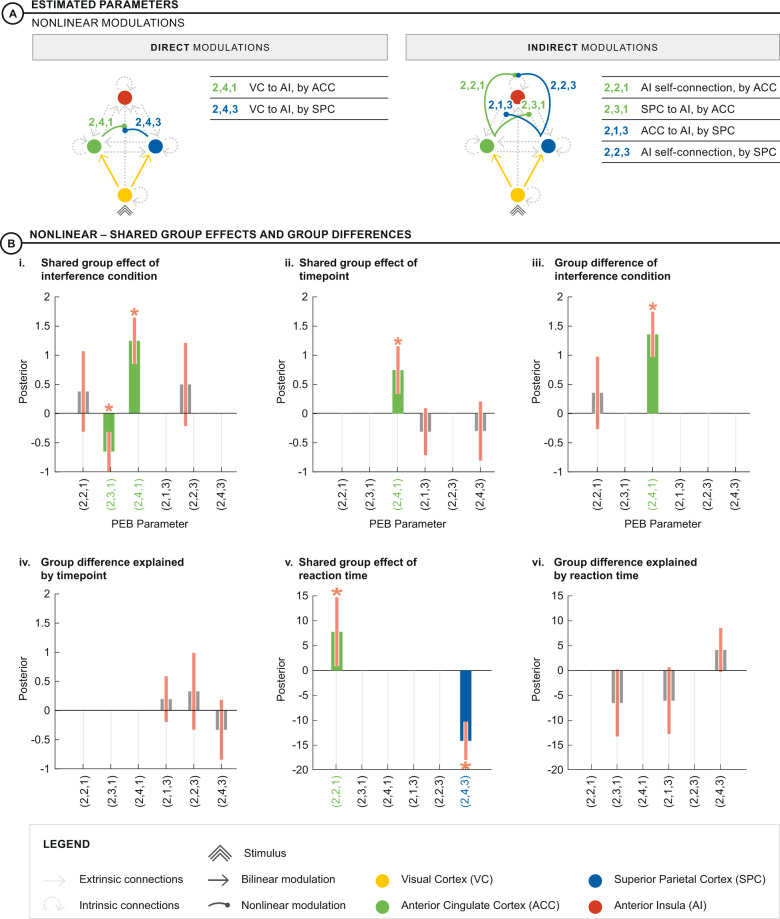
DCM parameters. **A** Parameters estimated for direct and indirect nonlinear modulation of stimulus conflict on connections to the AI. Green parameters show nonlinear modulations by ACC and blue parameters show nonlinear modulations by SPC. Direct nonlinear modulations gate input from VC to the AI. Indirect modulations require an initial bilinear, additive modulation to the ACC or SPC (yellow) before being gated by the parallel region. **B** Shared group effects and group differences: (i) the interference condition decreased indirect nonlinear modulation from SPC to AI, gated by the ACC (Parameter (2,3,1)), and increased the direct nonlinear modulation from VC to AI, also gated by the ACC (Parameter (2,4,1)) for both groups. (ii) Between timepoints both groups shared an increased direct nonlinear modulation of VC to AI, gated by the ACC (Parameter (2,4,1)). (iii) Overall, the HC group had increased direct nonlinear modulation from VC to AI, gated by ACC (parameter (2,4,3)), relative to the EP group. (iv) There were no group differences explained by timepoint. (v) Slower reaction times were associated with indirect nonlinear modulation of the AI self-connection, gated by the ACC (parameter (2,2,1)). Faster reaction times were associated with direct nonlinear modulation from VC to AI, gated by SPC (parameter (2,4,3)), for both groups. (vi) There were no group differences explained by reaction time. * indicates parameters where 90% credible interval does not reach or cross zero.

We adopted this strategy to permit group comparisons of the same parameters using Parametric Empirical Bayes (PEB), a hierarchical linear regression model that supports inference on DCM parameters [41]. The individual nonlinear and bilinear DCMs from each participant comprise the first level of the hierarchy. The connectivity parameters of these DCMs comprise the second level for each group. To examine longitudinal changes in effective connectivity we used regressors for shared effects [1] and timepoint differences [-1 baseline, 1 follow-up] (Fig. S1A). To examine effective connectivity associated with reaction time we used regressors for reaction time delta (mean centered) and its longitudinal differences [-1 baseline, 1 follow-up] (Fig. S1B). Group effects were then modeled at a third level PEB with shared [EP group 1; HC group 1] and group difference [EP group 1; HC group 1] regressors (Fig. S1C). Thus, third level PEBs examined group differences in effective connectivity and two-way interactions (i.e., group-by-timepoint and group-by-reaction time). PEBs for bilinear and nonlinear models were estimated separately. According to our baseline study [25], indirect nonlinear modulations (requiring an additive, linear modulation before input to the AI is gated by a third brain region) are representative of less efficient, tonic gain control whilst direct nonlinear modulations (input from VC to AI is gated by a third brain region) reflected more efficient, phasic gain control [25] (Fig. 3A).

Full details of participants, MRI data acquisition, pre-processing, task-related fMRI modeling is provided in the supplement.

## Results

### Demographics, clinical factors, and task performance

All diagnoses in the EP group did not change at follow-up. The EP and HC groups did not significantly differ in sex, age, substance use, IQ, or years of education at baseline and follow-up (Table 1). Mean chlorpromazine equivalent doses were not statistically different in the EP group between baseline (131.5 mg) and follow-up (144.7 mg). The EP group demonstrated a significant decrease in PANSS (paired *t*-test *t* = 2.706, df = 18, *p* = 0.015) (Table 1 and Fig. S2A) and significant increase in SOFAS (social and occupational functioning) (paired *t*-test, *t* = –4.609, df = 18, *p* = <0.001) (Table 1 and Fig. S2B), indicating clinical improvements in symptoms and social-occupational recovery.

When performing the MSIT, EP participants had significantly larger reaction time delta (interference condition reaction time minus neutral condition reaction time) compared to HC at baseline (independent sample *t*-test, *t* = 2.027, df = 35, *p* = 0.050), but not at follow-up (independent sample *t*-test, *t* = 0.992, df = 35, *p* = 0.328). There was a significant decrease in reaction time delta over timepoints for EP participants (Wilcoxon signed-rank, *W* = 188.000, df = 18, *p* = <0.001), but not HC (paired *t*-test, *t* = 1.191, df = 18, *p* = 0.249). However, the group-by-timepoint interaction in reaction time delta was not statistically significant (simple mixed ANOVA, *F* = 1.915, df = 36, *p* = 0.175) (Fig. S3). Changes in reaction delta amongst the EP participants correlated significantly with increases in SOFAS (rmcorr = –0.526, df = 18, *p* = 0.017) but not with changes in PANSS for (rmcorr = 0.429, df = 18, *p* = 0.059) when controlled for timepoint. *t*-tests and ANOVA met assumptions for normality and equal variances.

### Interference condition, group and timepoint: effects on neural activations

Significant effects of stimulus conflict (i.e., interference condition > neutral condition) were expressed in canonical CCS regions, including bilateral SPC, bilateral supplementary motor areas (confluent to the ACC), bilateral AI and right DLPFC (all *p* < 0.05 FWE) (Fig. 1B and online supplement Table S2). Significant effects of stimulus conflict also occurred in the bilateral VC (*p* < 0.05 FWE). There were no significant clusters for main effect of group and timepoint. However, an interaction effect of group-by-timepoint was expressed in a single, contiguous cluster that extended from the left superior occipital cortex into the precuneus and the SPC (*p* = 0.006 FWE cluster-corrected) (Fig. 1B and online Supplement Table S2).

### Interference condition, group, timepoint and reaction time: effects on effective connectivity

We next used variational Bayes to estimate the full bilinear and nonlinear DCM models (Fig. 2A), and PEB to identify task-modulated changes in effective connectivity common to both groups (“shared effects”), as well as timepoint, reaction time and group differences (Fig. 3). Here, we comment only on parameters where the 90% credible interval bars do not reach or cross zero. Among the bilinear modulations, there were no shared effects or group differences. In contrast, among the nonlinear modulations, there were several shared effects and group differences:

1. Interference condition, timepoint and group-by-timepoint (Fig. 3Bi–iv):
  The interference condition induced a decrease in the indirect nonlinear modulation of SPC to AI, gated by ACC (Fig. 3Bi; parameter (2,3,1)) and an increase in the direct nonlinear modulation, from VC to AI, also gated by the ACC (Fig. 3Bi; parameter (2,4,1)). The effect of timepoint, shared across groups, was associated with an increase in the direct nonlinear modulation of VC to AI, gated by the ACC (Fig. 3Bii; parameter (2,4,1) Pp > 99.9%). The effect of group revealed an increase in the same nonlinear parameter in the HC relative to the EP group (parameter (2,4,1) Pp > 99.9%; Fig. 3Biii). Therefore, although the HC group showed a stronger direct modulation of VC to AI, gated by the ACC, relative to EP, both groups contributed to a main effect of timepoint in this direct nonlinear modulation. There was no group-by-timepoint interaction (Fig. 3Biv).
2. Reaction time and group-by-reaction time (Fig. 3Bv–vi):
  We next used a third level PEB to study group and timepoint effects on reaction time, noting that a positive covariance indicates an association with increased reaction time delta (i.e., slower reaction times in interference compared to neutral tasks) and conversely negative covariance indicates an association with decreased reaction time delta. There was a positive covariance of reaction time delta with the indirect nonlinear modulation on the AI self-connection, gated by ACC (Fig. 3Bv; parameter (2,2,1) Pp 66%). Notably, there was a strongly negative covariance of reaction time delta with the direct nonlinear modulation of VC to AI, gated by SPC (Fig. 3Bv; parameter (2,4,3) Pp > 99.9%). There was no group-by-reaction time interaction (Fig. 3Bvi).
3. Comparison of bilinear and nonlinear models
  We also compared the log evidence between each bilinear and nonlinear third level PEB to examine for best explanation of estimated parameters. The nonlinear third level PEBs for effect of group-by-timepoint and group-by-reaction time showed better model evidence (see supplementary information).

### Within EP group post hoc analysis

By follow-up, EP participants showed significantly less symptoms and improved social-occupational functioning, faster reaction times, and a normalization of SPC activation relative to the HC group. Moreover, improvements in social-occupational functioning correlated with reduced reaction time delta. Finally, we noted that both groups contributed to the shared effect of interference condition decreasing an indirect nonlinear modulation of SPC to AI, as gated by ACC (Fig. 3Bi; parameter (2,3,1)); both groups also contributed to the main effect of timepoint with an increase in the direct nonlinear modulation of VC to AI, as gated by ACC (Fig. 3Bii; parameter (2,4,1)). We therefore employed post hoc analysis of effective connectivity within the EP group. We derived effects of the interference condition, timepoint and reaction time, to explore how the EP group contributed to the shared group effects, noting the absence of significant group-by-timepoint effects as a caveat. To examine effective connectivity changes explained by timepoint, we performed an automatic search of reduced PEB models of the already estimated second level PEB for the EP group. To examine how reaction time changed with timepoint we estimated second level PEBs with reaction time delta as the covariate of interest for baseline and follow-up, respectively (Fig. S1E). The interaction between reaction time delta and timepoint was then examined at the third level (Fig. S1F).

**Fig. 4:**
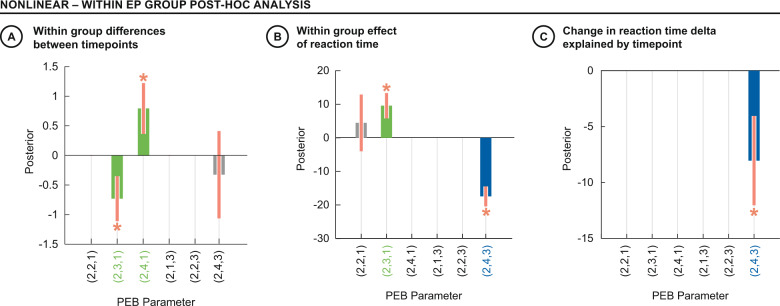
DCM parameters: Within EP group post-hoc analysis. Within EP group post hoc analysis. Green parameters show nonlinear modulations by ACC and blue parameters show nonlinear modulations by SPC. **A** Between timepoints, the indirect nonlinear modulation from SPC to AI, gated by the ACC decreased (Parameter (2,3,1)). In contrast, the direct nonlinear modulation from VC to the AI, gated by the ACC, increased with timepoint (Parameter (2,4,1)). **B** Slower reaction times were associated with stronger indirect nonlinear modulation of AI, gated by ACC (Parameter (2,3,1)); Faster reaction times were associated with stronger direct nonlinear modulation of AI, gated by SPC (Parameter (2,4,3)). **C** Improvement in reaction time delta (i.e., smaller values) was associated with stronger direct nonlinear modulation of AI, gated by SPC, between timepoints, as indicated by a negative covariance (Parameter (2,4,3). * indicates parameters where 90% credible interval does not reach or cross zero.

Within the EP group, we observed an increase in the direct nonlinear modulation of VC to AI, gated by ACC (Fig. 4A parameter (2,4,1) Pp > 99.9%) and a decrease in the indirect nonlinear modulation of SPC to AI, gated by ACC (Fig. 4A parameter (2,3,1) Pp > 99.9%) between timepoints. Faster reaction time was associated with direct nonlinear modulation of VC to AI, as gated by SPC, as a main effect (Fig. 4B parameter (2,4,3), Pp > 99.9%). Furthermore, stronger direct nonlinear modulation of VC to AI, as gated by SPC, was associated with improved reaction time delta from baseline to follow-up timepoint (Fig. 4C parameter (2,4,3), Pp 98.8%).

### Post hoc model parameter correlation with recovery measures

We conducted univariate correlation of bilinear and nonlinear parameters to assess their relationship with clinical measures of recovery in the EP group. None of the nonlinear parameters showed significant repeated measures correlation with changes in symptoms or social-occupational functioning over timepoints.

## Discussion

Cognitive recovery following a psychotic episode is key to positive social and occupational outcomes. We observed improved symptoms, social-occupational function, task fMRI responses, and reaction times to stimulus conflict in an EP cohort 12 months after an initial baseline assessment. Analysis of basic task fMRI activations suggested a normalization of the SPC response to conflict, which is notable in the context of improved cognitive function in the EP cohort, given the contribution of the SPC to cognitive control. Computational modeling of these data suggested that faster reaction times to stimulus conflict was dependent on direct gating of sensory input to the AI by the SPC. Within the EP group, stronger direct nonlinear modulation of this connection at follow-up was associated with improvement in reaction time, which co-occurred with the transition from indirect to direct nonlinear modulation of the AI by the ACC. These findings suggest that the parsimonious integration of cingulo-opercular and fronto-parietal processing of stimulus conflict facilitates the improved task performance seen in the EP group. Despite initial differences at baseline, EP participants adopted more direct models of processing stimulus conflict, like HCs, at follow-up, which may be consistent with a normalizing effect of treatment and recovery.

Deficits of functional integration between the salience and central executive networks are known to underlie symptoms and cognitive impairment in EP [19–22]. Here, we used a task that engages cognitive control, the MSIT, to evaluate how cingulo-opercular (i.e., salience) and fronto-parietal (i.e., central executive) networks functionally integrate. Our longitudinal findings emphasize how improved cognitive control depends on a reorganization of the integration of cingulo-opercular and fronto-parietal networks. For example, a direct gating of sensory input to the AI by the SPC was associated with faster reaction times in both groups, consistent with the role of fronto-parietal networks in context-specific performance control [12]. Furthermore, the hierarchical gating role of the ACC in both groups accords with the known role of ACC in maintaining and updating task context to the AI, particularly as task demands escalate under the challenge of stimulus conflict [42].

Hierarchical gating of inputs depends upon neuromodulatory processes that enable rapid changes in effective connectivity at the neuronal level, captured by nonlinear modulation in DCM [18]. Nonlinear modulations represent effects of short-term plasticity that alter synaptic strengths at time scales of milliseconds to minutes [43], reflective of neuronal gain control [44]. Of particular relevance is the short-term plasticity induced by NMDA receptors, since NMDA hypofunction is central to the disconnection hypothesis of schizophrenia [39]. We suggest that the longitudinal transition from indirect to direct nonlinear modulation by the EP participants may represent remediation of neuronal level processes underlying gain control. This hypothesis is supported by a recent demonstration of how gain control at the neuronal microscale translates to neuronal population dynamics at the large-scale network level to support cognitive function [33].

These findings add to a growing literature that addresses the neurobiology of cognitive deficits in psychotic disorders, particularly within the context of gain control abnormalities. Excitatory-inhibitory imbalance of cortical circuits is thought to underlie cognitive impairments, not only in psychotic disorders, but mental health conditions in general [45]. Gain control deficits provide a plausible mechanism by which this excitatory-inhibitory imbalance may arise due to altered post-synaptic sensitivity to pre-synaptic inputs [46, 47]. Recent work using DCM to integrate multimodal EEG and fMRI across multiple resting state and task paradigms suggests that psychotic symptoms may be related to increased self-inhibition on cortical pyramidal cells (i.e., reduced synaptic gain) as a compensatory mechanism to restore excitatory-inhibitory imbalance [26]. Our work here is limited to observing brain region changes of excitability only. This is inherent to the coarse temporal limitation of fMRI, compared to EEG, which allows inference on microcircuit changes on a millisecond scale, hence allowing for more specific parameterization of gain. Secondly, nonlinear DCM for fMRI, as used in our study, cannot completely account for nonlinearities arising from hidden neuronal and physiological states [18] and cannot model neural noise, as is performed in stochastic DCM [48]. Nonetheless, our work demonstrates how gain deficits may play out across large-scale networks, where the CCS-mediated gain control increases sensitivity to salient stimuli and down regulates noisy inputs. Our findings also suggest that gain control deficits, and accompanying symptoms such as cognitive impairment, may potentially be remediable at a relatively early stage of the disorder.

There are several limitations that warrant discussion. First, the inevitable loss of participants to follow-up impacted on the sensitivity of DCM parameter estimations and decreased the power to detect accompanying brain-behavior effects. However, we did observe the expected clinical and social-occupational functioning improvements in the EP cohort, as well as an interaction of group-by-timepoint in the SPC. The group differential effect in the SPC activation over timepoints was not explicitly modeled, due to our a priori hypothesis, which may have yielded brain-behavior interaction effects in the DCM. Nonetheless, computational modeling approaches based on relative posterior evidence, such as DCM and PEB, permit a flexible and valid exploration of study effects without being subject to the “all-or-nothing” constraints of classical statistical inference. Second, restricting the analyses to exclusively left-side cortical regions is not fully representative of how the CCS processes stimulus conflict. In addition, we did not include the DLPFC, a core node in the fronto-parietal network consistently implicated in studies of cognitive control. However, there was no left-sided DLPFC engagement in the interference condition at either baseline or follow-up. The presence of a strong right-sided DLPFC activation suggests that inter-hemispheric models of stimulus conflict processing during the MSIT may be more generalizable. However, the choice to constrain to a left-hemispheric analysis is further justified by the group-by-timepoint interaction in task activation of the left SPC. Furthermore, the longitudinal study was motivated by a well-defined hypothesis from the baseline study that group differences in stimulus conflict processing were mediated by a left-sided group differential effect in the AI. Finally, we could not control for the effect of substance use in participants, nor regress it as a nuisance covariate as its rating did not accurately reflect the pattern of use (i.e., number of substances used or the composite amount used).

We also note that the post hoc within EP group analyses were performed in the absence of interaction effects of effective connectivity at the group level. However, measures of symptomatic and functional recovery in the clinic cannot be benchmarked against a HC cohort, but only relative to each patient’s baseline. Since the EP group demonstrated significant longitudinal reductions in symptoms and reaction time, as well as increase in social-occupational functioning, corresponding longitudinal changes in effective connectivity may provide additional insights into these indices of clinical recovery. For example, the longitudinal improvement in reaction time in the EP group was associated with increased direct nonlinear modulation of VC to the AI, by the SPC. This underscores the notion that behavioral efficiency depends on the flexibility of the cingulo-opercular and fronto-parietal networks to permit more direct processing of sensory inputs, and our findings suggest that the EP group were able to reconfigure this over timepoints.

Employing simple, robust paradigms, such as the strong neural effects induced by stimulus conflict in the MSIT, should be considered in the endeavor to explore the mechanisms underlying symptomatic, cognitive, and behavioral dimensions of psychotic disorders. Although there is a trend towards modeling specific dimensions of psychosis using bespoke and complex tasks paradigms [49], we believe that the value of simple tasks, such as the MSIT, is the relative ease by which these tasks can be deployed in large multi-center, multi-site studies with longitudinal designs. These are needed to understand the neurobiological mechanisms underpinning the symptomatic and functional trajectories seen in individuals with EP.

## Supplementary information

Supplement

## Data Availability

Access to raw data from this study is subject to approval from the local HREC who gave approval for this study and is available on request. The analysis packages used in this study is available through: fMRIPrep: https://github.com/nipreps/fmriprep, Statistical Parametric Mapping: https://www.fil.ion.ucl.ac.uk/spm/software/.

## Code availability

Code used for analysis is available at https://github.com/bjornburg/Longitudinal-changes-in-gain-and-cognition-in-early-psychosis.
