# Supplementary material for: Longitudinal changes in neural gain and its relationship to cognitive control trajectory in young adults with early psychosis
Source: Transl Psychiatry. 2023 Mar 3;13:77. doi: 10.1038/s41398-023-02381-x (PMC9981770; doi:10.1038/s41398-023-02381-x)
Supplement: Supplementary file 1 — Supplement [file 41398_2023_2381_MOESM1_ESM.docx]

**Longitudinal changes in neural gain and its relationship to cognitive control trajectory in young adults with early psychosis**

***Supplementary Information***

**Contents**

Supplementary Methods

Supplementary Results

Supplementary Fig. S1-S11

Supplementary Table S1, S2

Supplementary References

**Methods**

Participants

EP participants had experienced a first episode of psychosis in the last two years and met the following eligibility criteria; (i) normal pre-morbid functioning, as rated by the Diagnostic Interview for Psychoses (DIP) (1), (ii) absence of childhood neurodevelopmental or organic brain disorder, and (iii) onset of psychosis after the age of sixteen. These criteria ensured EP participants were likely to have had relatively normative neurodevelopment until psychosis onset, since childhood onset psychoses are associated with more severe deficits.

Participants consented to be contacted at 12 months after their baseline assessment. All participants who followed up completed their assessment within the twelfth month after the baseline timepoint. Participants lost to follow-up either declined or were not contactable for a follow-up assessment.

fMRI paradigm

To investigate the brain and behavioral effects of interference on the cognitive control system (CCS) the multisource interference task (MSIT) (2) was used during fMRI. The task uses visual stimuli comprising a set of 3 numbers (either “0”, “1”, “2” or “3”) that appear in center of the screen for approximately 2.5 seconds, with one number always being unique from the other two (i.e., distractors). Participants were instructed to select the identity of the unique number by button press “as quickly as possible, but do not sacrifice accuracy for speed”. 12 stimuli were contained in alternating 45 second blocks of neutral and interference condition trials, interspersed by 15 second periods of visual fixation. In total, participants undertook 4 blocks of neutral and 4 blocks of interference for a total of 96 trials. During neutral condition the unique number (“1”, “2”, “3”) was surrounded by “0”s and always located in its spatially correct position ( “1 0 0”, “ 0 2 0”, “0 0 3”). During interference condition, the unique number (“1”, “2”, “3”) was flanked by distracting numbers e.g. (e.g. “1 3 1”) to produce a flanker effect(3). Additionally, the unique number was never in its correct spatial position (e.g., “3 1 3”), thereby producing a spatial incongruence effect. Accuracy and reaction time during trials were acquired to measure the effect of interference. All participants undertook a practice session on a laptop outside of the MRI scanner. This session involved one block of neutral and one block of interference trials. Once participants understood the task, they undertook the full task in the scanner.

MRI data acquisition

T2*-weighted echoplanar images (EPI) were acquired in a posterior-anterior (PA) phase encoding direction, consisting of 60 slices of 2.4mm thickness in interleaved order, obtained with a repetition time (TR) = 82 ms, echo time (TE) = 3.3ms, flip angle = 53 degrees, field of view (FOV) = 206mm x 206mm, in-plane resolution = 86 x 86 and acceleration factor of 6. Spin echo field maps were obtained in PA and anterior-posterior (AP) directions, containing 60 slices with a TR = 6350ms and TE = 63ms. T1-weighted (T1w) MPRage images consisted of 208 slices of 0.8mm slice thickness in interleaved order, with a TR = 2400ms, TE = 2.22 ms, flip angle = 8 degrees, FOV = 256mm x 256mm.

In terms of framewise displacement, there were no significant differences between group (F = 1.177, df = 36, p = 0.285) and timepoint (F = 0.085, df = 36, p = 0.772) and no group-by-timepoint interaction (F = 0.294, df = 36, p = 0.591). One HC participant was removed from the analysis at baseline due to image artefact.

Task fMRI pre-processing

Task data were pre-processed with fmriprep (version 20.0.6) (4)and an in house (fmripop; RRID:SCR_001362) Nipype based tool (5) was used to regress fMRI confounds of motion, cerebrospinal fluid (CSF) and white matter(WM)), smooth data (full half width maximum 8mm) and censor volumes from the pre-processed outputs.

As quoted verbatim from fmriprep pre-processing output:

The T1-weighted (T1w) image was corrected for intensity non-uniformity (INU) with N4BiasFieldCorrection (6), distributed with ANTs 2.2.0 ((7); RRID:SCR_004757), and used as T1w-reference throughout the workflow. The T1w-reference was then skull-stripped with a Nipype implementation of the antsBrainExtraction.sh workflow (from ANTs), using OASIS30ANTs as target template. Brain tissue segmentation of cerebrospinal fluid (CSF), white-matter (WM) and gray-matter (GM) was performed on the brain-extracted T1w using fast (FSL 5.0.9, RRID:SCR_002823,((8)). Brain surfaces were reconstructed using recon-all (FreeSurfer 6.0.1, RRID:SCR_001847,(9)), and the brain mask estimated previously was refined with a custom variation of the method to reconcile ANTs-derived and FreeSurfer-derived segmentations of the cortical gray-matter of Mindboggle (RRID:SCR_002438,(10)). Volume-based spatial normalization to one standard space (MNI152NLin6Asym) was performed through nonlinear registration with antsRegistration (ANTs 2.2.0), using brain-extracted versions of both T1w reference and the T1w template. The following templates were selected for spatial normalization: FSL’s MNI ICBM 152 non-linear 6th Generation Asymmetric Average Brain Stereotaxic Registration Model [(11), RRID:SCR_002823; TemplateFlow ID: MNI152NLin6Asym].

For each of the 1 BOLD runs found per subject (across all tasks and sessions), the following preprocessing was performed. First, a reference volume and its skull-stripped version were generated using a custom methodology of fMRIPrep. A B0-nonuniformity map (or fieldmap) was estimated based on two (or more) echo-planar imaging (EPI) references with opposing phase-encoding directions, with 3dQwarp (12)(AFNI 20160207). Based on the estimated susceptibility distortion, a corrected EPI (echo-planar imaging) reference was calculated for a more accurate co-registration with the anatomical reference. The BOLD reference was then co-registered to the T1w reference using bbregister (FreeSurfer) which implements boundary-based registration (13). Co-registration was configured with six degrees of freedom. Head-motion parameters with respect to the BOLD reference (transformation matrices, and six corresponding rotation and translation parameters) are estimated before any spatiotemporal filtering using mcflirt (FSL 5.0.9,(14)). BOLD runs were slice-time corrected using 3dTshift from AFNI 20160207 ((12), RRID:SCR_005927). The BOLD time-series (including slice-timing correction when applied) were resampled onto their original, native space by applying a single, composite transform to correct for head-motion and susceptibility distortions. These resampled BOLD time-series will be referred to as preprocessed BOLD in original space, or just preprocessed BOLD. The BOLD time-series were resampled into several standard spaces, correspondingly generating the following spatially-normalized, preprocessed BOLD runs: MNI152NLin6Asym. First, a reference volume and its skull-stripped version were generated using a custom methodology of fMRIPrep. Several confounding time-series were calculated based on the preprocessed BOLD: framewise displacement (FD), DVARS and three region-wise global signals. FD and DVARS are calculated for each functional run, both using their implementations in Nipype (15). The three global signals are extracted within the CSF, the WM, and the whole-brain masks. Additionally, a set of physiological regressors were extracted to allow for component-based noise correction (CompCor,(16)). Principal components are estimated after high-pass filtering the preprocessed BOLDtime-series (using a discrete cosine filter with 128s cut-off) for the two CompCor variants: temporal (tCompCor) and anatomical (aCompCor). tCompCor components are then calculated from the top 5% variable voxels within a mask covering the subcortical regions. This subcortical mask is obtained by heavily eroding the brain mask, which ensures it does not include cortical GM regions. For aCompCor, components are calculated within the intersection of the aforementioned mask and the union of CSF and WM masks calculated in T1w space, after their projection to the native space of each functional run (using the inverse BOLD-to-T1w transformation). Components are also calculated separately within the WM and CSF masks. For each CompCor decomposition, the k components with the largest singular values are retained, such that the retained components’ time series are sufficient to explain 50 percent of variance across the nuisance mask (CSF, WM, combined, or temporal). The remaining components are dropped from consideration. The head-motion estimates calculated in the correction step were also placed within the corresponding confounds file. The confound time series derived from head motion estimates and global signals were expanded with the inclusion of temporal derivatives and quadratic terms for each (17). Frames that exceeded a threshold of 0.5 mm FD or 1.5 standardised DVARS were annotated as motion outliers. All resamplings can be performed with a single interpolation step by composing all the pertinent transformations (i.e. head-motion transform matrices, susceptibility distortion correction when available, and co-registrations to anatomical and output spaces). Gridded (volumetric) resamplings were performed using antsApplyTransforms (ANTs), configured with Lanczos interpolation to minimize the smoothing effects of other kernels (18). Non-gridded (surface) resamplings were performed using mri_vol2surf(FreeSurfer).

Many internal operations of fMRIPrep use Nilearn 0.6.2 ((19), RRID:SCR_001362), mostly within the functional processing workflow.

Task-related fMRI modelling

At the first level, t-contrasts images (interference > neutral condition) were obtained in each subject at both timepoints. At the second level, t-contrast images were used to conduct within-subject, paired t-tests (baseline > follow-up and baseline < follow-up) and a t-test (baseline + follow-up) for each subject at both timepoints. The main effect of group was examined by conducting unpaired t-tests of the within subject one t-test contrasts (i.e., EP group > HC group and EP group < HC group). The main effect of time was examined by conducting t-tests of the within subject paired t-tests (i.e., baseline > follow-up and baseline < follow-up). The interaction of group-by-time was examined by conducting unpaired t-tests of the within subject paired t-tests (i.e., baseline > follow-up and baseline < follow-up).

Dynamic causal modelling specification

Each DCM was estimated by extracting the first eigenvariate from a five-millimeter radius sphere volume of interest (VOI) from the F-contrast images of the GLM. VOIs were representative of left-sided brain nodes from the AI, ACC, SPC and the VC. Within subject co-ordinates of the VOIs from the baseline timepoint were used as a mask, and the first eigenvariate was extracted from the local maxima at the follow-up timepoint. This ensured that each region was represented by the same VOI across both timepoints.

For DCM matrix specification see <https://github.com/bjornburg/Longitudinal-changes-in-gain-and-cognition-in-early-psychosis>.

Bayesian Model Averaging

To examine which parameters contributed most model evidence we applied an automatic search, which evaluated for evidence for large numbers of reduced PEB models with mixtures of parameters switched “off”. Parameters from the best reduced models during this search were averaged (i.e., the Bayesian model average). To capture differences in gain control efficiency, we were specifically interested in parameters involving gating modulations (i.e., nonlinear).

**Results**

Clinical factors

Six EP participants were on lithium instead of antipsychotic at baseline, with five participants on lithium by follow-up. Five EP participants were on combination antipsychotic/antidepressant or lithium/antidepressant at baseline, while four participants were on combination medications at follow-up.

There were no significant baseline clinical differences between EP participants who were followed-up and who were lost to follow-up as measured by the PANSS (paired t-test, t = 1.581, df = 28, p = 0.1251) and SOFAS (paired t-test, t = 0.4266, df = 28, p = 0.6279) (Supplementary Table S1).

There was no significant difference at baseline between EP participants or HC participants who were followed-up and who were lost to follow-up in terms of reaction time delta (independent t tests, t = 0.6876, df = 28, p = 0.4974 and t = 1.229, df = 27, p = 0.2297, respectively) (online supplement table S1).

Shared group effects and group differences in nonlinear parameters

Since the effect of group and timepoint were parsimoniously modelled in the third level nonlinear group-by-timepoint PEB (Fig. 3A i-iv.) we did not comment on the shared group effects and group differences in the third level nonlinear group-by-reaction time in the manuscript. Please Fig. S4 to see these results.

In the within-EP group second level nonlinear PEB examining effect of reaction time there was an effect of timepoint (modelled as the second covariate) that was not commented on in the manuscript, since this was parsimoniously modelled in the within-EP group second level nonlinear PEB examining effect of timepoint. This re-confirmed the effect of increase in direct nonlinear modulation of VC to AI, as gated by ACC (parameter 15, Pp >99.9%) (Fig. S5).

There were several within HC group findings for the nonlinear second level PEBs examining effect of timepoint and reaction time. However, we did not comment on these results since the absence of interaction effects in the third level PEBs did not provide justification to examine these results. Please see Fig. S6 & S7.

Group and within-group shared effects and differences in bilinear parameters

As described in the manuscript, there were no shared group effects or group differences in the third level bilinear PEBs for effect of group-by-timepoint or group-by-reaction time.

However, there were within group shared effects and within group differences as an effect of timepoint and effect of reaction time for both groups in the second level PEBs. There were also shared effects and differences when examining the interaction between reaction time delta and timepoint within the EP group. We did not comment on these results since the absence of group, timepoint and interaction effects in the third level bilinear PEBs did not provide justification to examine these results. Please see Fig. S8 – S11.

Comparison of PEB models

For all PEB-of-PEBs we conducted 2^nd^ Level and 3^rd^ Level approaches specified at <https://en.wikibooks.org/wiki/SPM/Parametric_Empirical_Bayes_(PEB)>. When comparing the free energy between both approaches (PEB.F) the third level approach was the favoured option.

We also compared the free energy of the third level PEB-of-PEBs between bilinear and nonlinear models. Using a default estimation of the third level bilinear and nonlinear PEBs for group-by-timepoint and group-by-reaction time, nonlinear models showed better evidence. This was achieved by subtracting the PEB.F value between the bilinear and nonlinear third level PEBs. However, there was an argument that to make the PEBs truly comparable between bilinear and nonlinear models, the bilinear models needed re-estimation with a nonlinear integrator. We performed this by re-estimating the bilinear models through SPM GUI with the nonlinear option turned “on” but with all D-matrix modulations set to “0”. Using this approach, the bilinear models showed better evidence. However, it also changed the results of the third level bilinear PEBs, inducing a common group effect of decreased self-inhibition in the anterior insula (Pp >99.9%) and a group difference of HC showing less self-inhibition relative to EPY (Pp >99.9%). We are uncertain as to why changing the integrator would produce different results for the bilinear models. Therefore, we only specified the results of the bilinear and nonlinear comparison using the default estimation of bilinear models in the manuscript.

**Supplementary tables and figures**

**Fig. S1 Design matrix for second level PEBs and third level PEB-of-PEBs for group and within Early Psychosis group comparisons**

1. **B) C)**


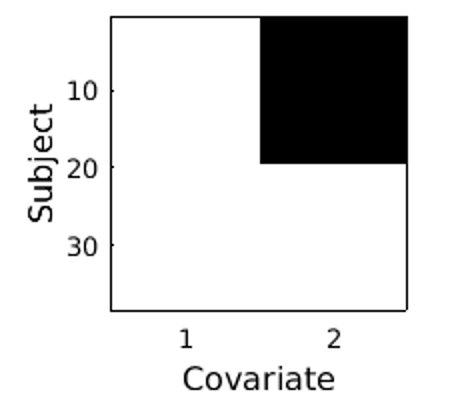

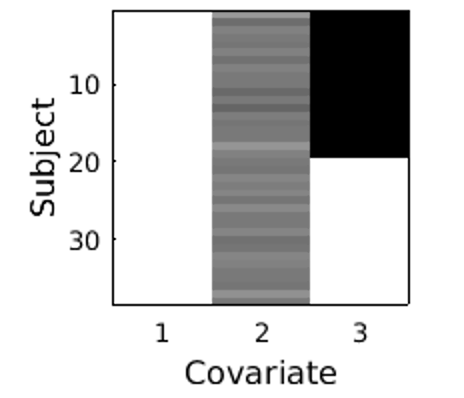

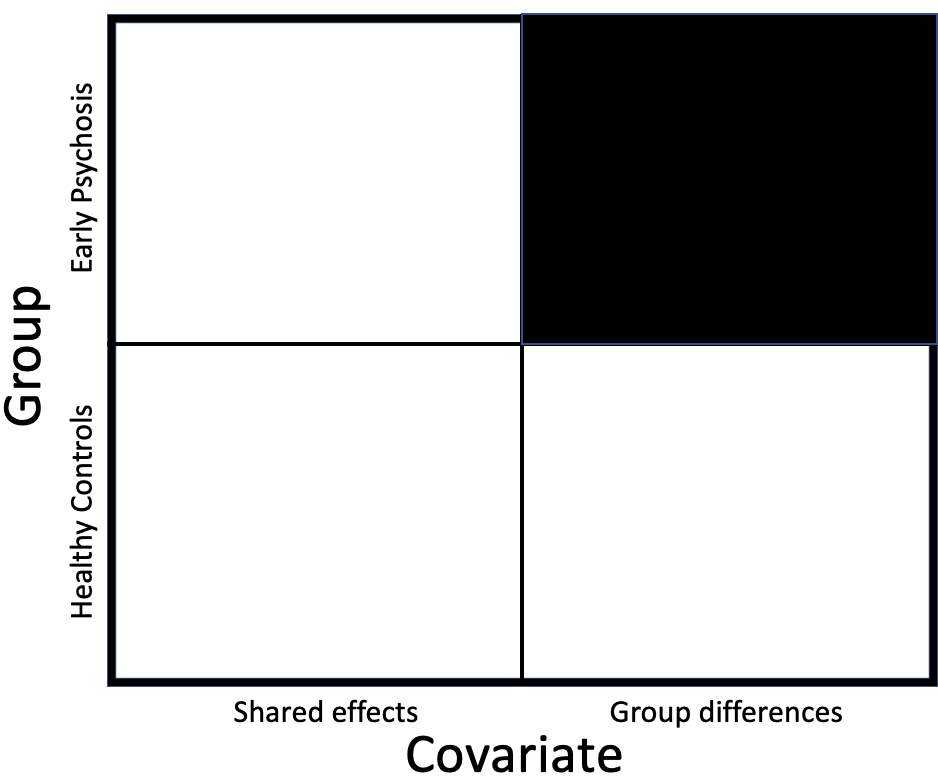


**D)**


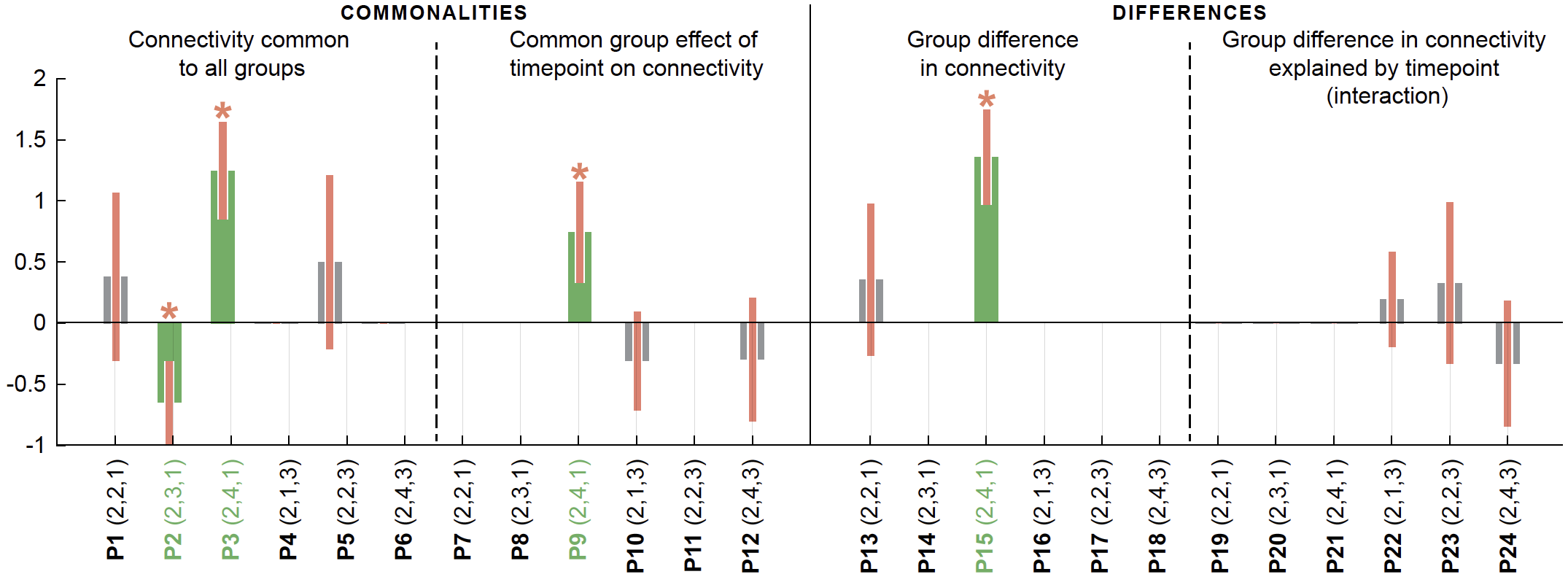


**E) F)**

**
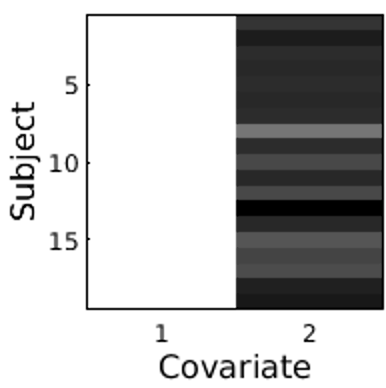

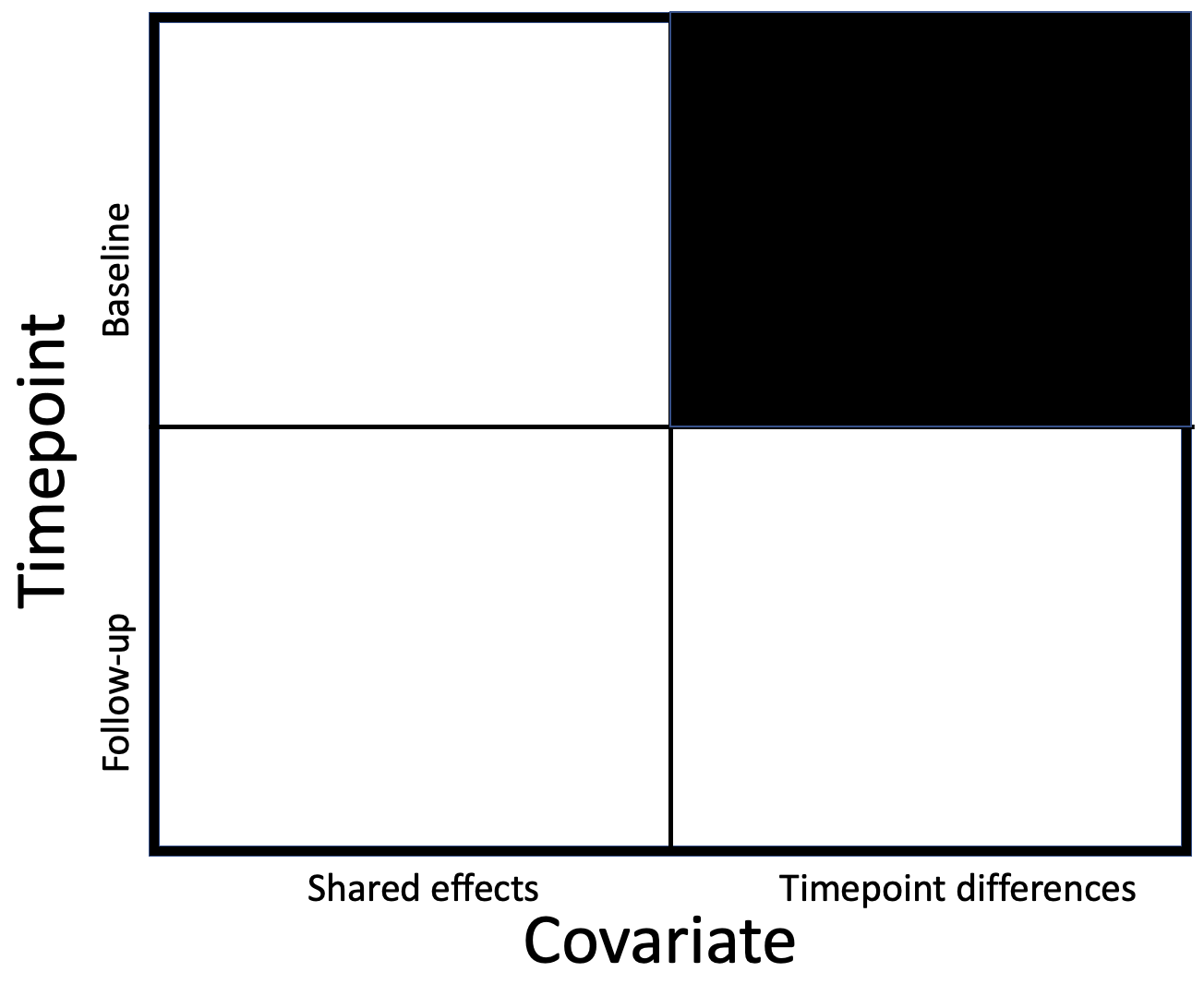
**

**G)**

**
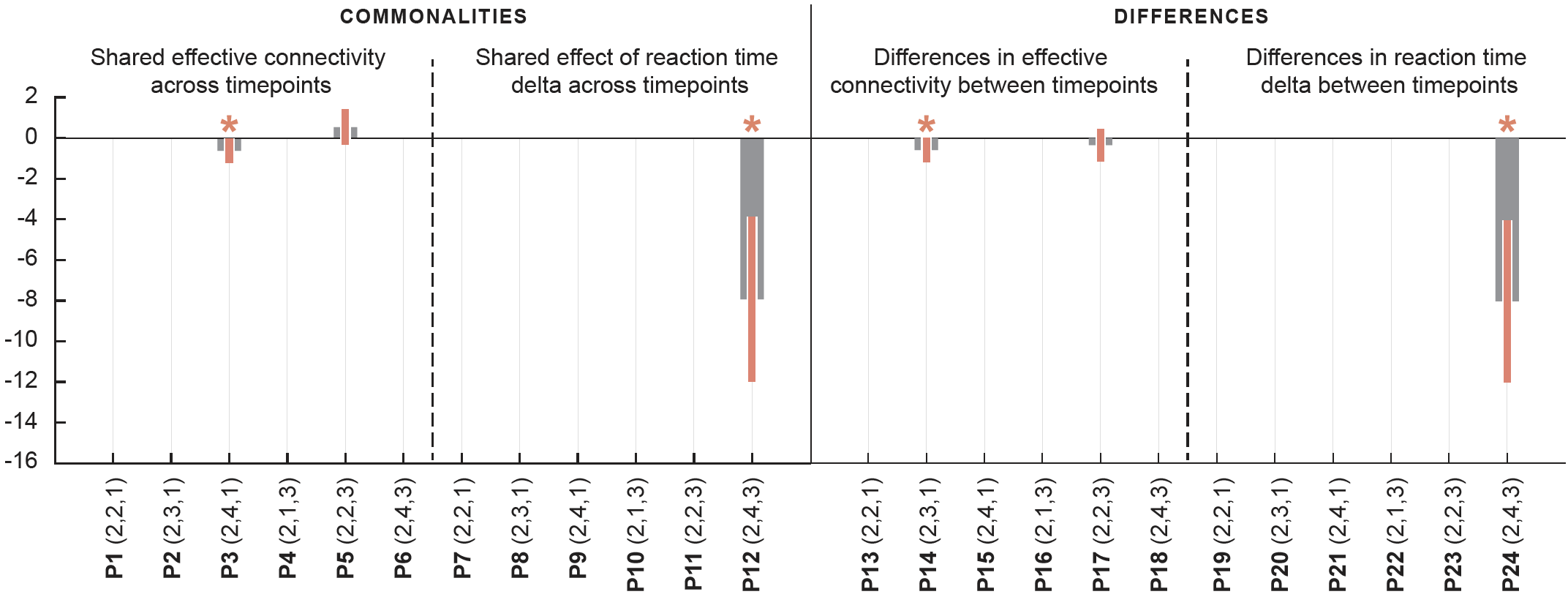
**

**A** Design matrix for each group’s second level PEBs modelling effect of timepoint, with shared effects in first column and timepoint in second column. **B** Design matrix for each group’s second level PEBs modelling effect of reaction time, with shared effects in the first column, reaction time delta in the second column and timepoint in third column. **C** Design matrix for third level PEB-of-PEBs modelling effect of group, with shared-effects in first column and group differences in second column. Second-level PEBs model within-group shared-effects and differences and are entered at the third level. **D** For example, the PEB output of design matrix C modelling the effect of timepoint shows effective connectivity shared by both groups in the first column, main effect of timepoint in the second column, group differences of effective connectivity in the third column, and interaction of group and timepoint in the fourth column **E** Design matrix for second level PEBs for the EP group modelling effect of reaction time, with shared effects in the first column and reaction time delta in the second column. A second level PEB was created for baseline and follow-up timepoints, respectively. **F** These second level PEBs were taken to the third level, with a design matrix modelling shared effects and differences of timepoint. **G** The PEB output of design matrix Fig. S1F is effective connectivity shared across timepoints in first column, main effect of reaction time delta in the second column, differences in effective connectivity between timepoints, and interaction of reaction time delta and timepoint in the fourth column.

**Fig. S2**

1. **Symptoms over time B) Functioning over time**


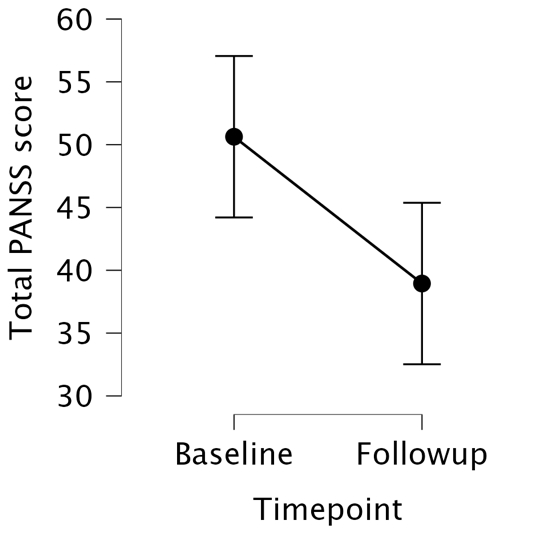

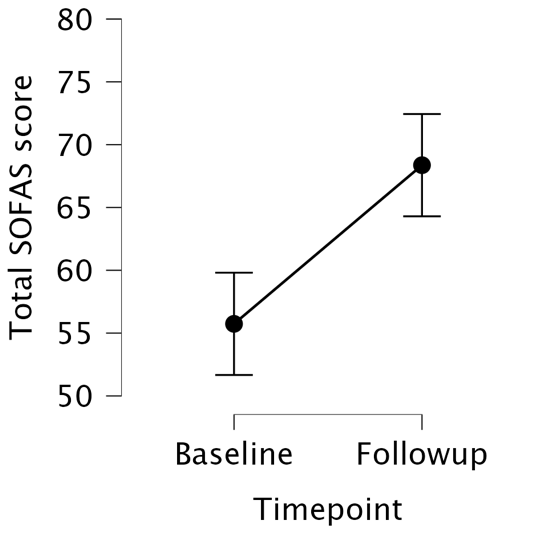


**A** EP group showed a significant reduction in global PANSS over time point, indicating symptomatic improvement over 12 months. **B** EP group showed a significant increase in SOFAS over time point, indicating social and occupational improvement over 12 months. Error bars show standard error (within subjects).

**Table S1**

**Comparison of Follow-up participants versus Lost to Follow-up participants**

|  | **Healthy Control Follow-up** | **Healthy Control Lost** | **Early Psychosis Follow-up** | **Early Psychosis Lost** |
| --- | --- | --- | --- | --- |
| **Characteristics** | **Mean (SD)** | | **Mean (SD)** | |
|  |  | |  | |
| Reaction time delta (seconds) | 0.324 (0.1005) | 0.372 (0.093) | 0.399 (0.124) | 0.435 (0.162) |
| PANSS |  |  | 50.6 (21.2) | 40.1 (7.8) |
| SOFAS |  |  | 55.7 (15.0) | 58.3 (16.8) |

There was no difference between participants in task performance (i.e., reaction time) in those that were followed-up and those who were lost to follow-up. There was no difference in EP participants in terms of symptoms and functioning in those who followed-up and those who were lost to follow-up.

**Fig. S3**

**Reaction time delta: group-by-timepoint**


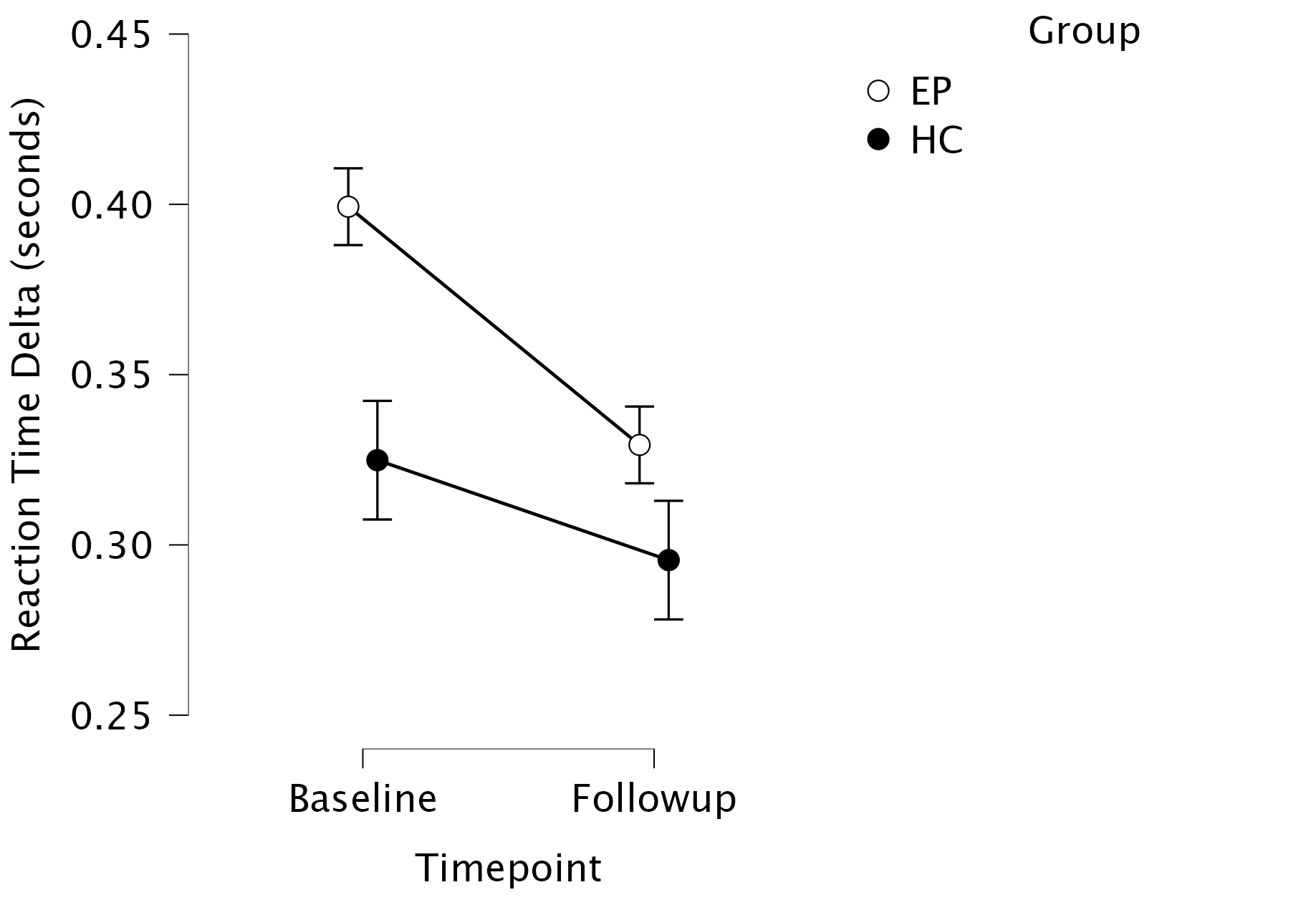


Despite a significant group difference in baseline time point reaction time that converges at follow-up, there was no significant interaction of group-by-timepoint. Error bars show standard error (within subjects).

**Table S2**

| **Cluster region** | **Peak co-ordinates**  **(x,y,z)** | **Peak Level t** | **Peak level z** | **Peak level P**  **(FWE corrected)** | **Cluster-level P**  **(FWE corrected)** | **Cluster size**  **(voxels)** |
| --- | --- | --- | --- | --- | --- | --- |
| **Effect of interference contrast over time points**  **Cluster forming height threshold at 0.05 FWE corrected** | | | | | | |
| **Right Visual Cortex** | 27 -97 -7 | 14.95 | Inf | <0.0001 | <0.0001 | 1025 |
| **Left Visual Cortex** | -33 -95 -7 | 14.57 | Inf | <0.0001 | <0.0001 | 1109 |
| **Left Superior Parietal Cortex** | -25 -66 48 | 12.95 | Inf | <0.0001 | <0.0001 | 2446 |
| **Right Superior Parietal Cortex** | 27 -69 48 | 11.93 | Inf | <0.0001 | <0.0001 | 1886 |
| **Left Supplementary Motor Area ^1^** | -47 3 34 | 11.32 | Inf | <0.0001 | <0.0001 | 3057 |
| **Cerebellum** | -1 -52 -22 | 10.28 | Inf | <0.0001 | <0.0001 | 1345 |
| **Left Anterior Insula** | -30 20 5 | 10.05 | Inf | <0.0001 | <0.0001 | 398 |
| **Right Anterior Insula** | 35 20 5 | 9.99 | Inf | <0.0001 | <0.0001 | 482 |
| **Right Supplementary Motor Area ^1^** | 47 6 31 | 9.26 | 7.49 | <0.0001 | <0.0001 | 282 |
| **Right Dorsolateral Prefrontal Cortex** | 39 32 24 | 7.29 | 6.29 | <0.0001 | <0.0001 | 295 |
| **Group-by-timepoint interaction**  **Cluster forming height threshold at 0.001 uncorrected** | | | | | | |
| - **Superior occipital cortex** - **Precuneus** - **Superior Parietal Cortex** | -18 -76 34  -11 -80 43  -6 -73 58 | 4.99  4.24  4.05 | 4.32  3.79  3.65 | 0.278  0.731  0.731 | 0.006 | 223 |

^1^ Anterior cingulate cortex clusters were confluent within bilateral supplementary motor area cluster.

**Fig. S4**


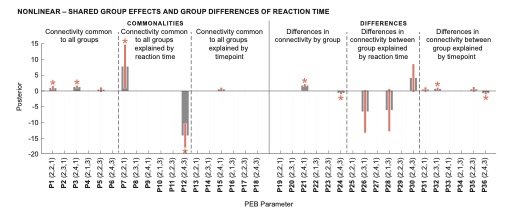


As stated in the manuscript, effects of group and timepoint were parsimoniously modelled. Here we comment on how modelling timepoint as the second covariate, with reaction time as the covariate of interest, changes some of the effects for group and timepoint. For the third level nonlinear group-by-reaction time PEB, modelling reaction time and timepoint changed several shared group effects and group differences compared to the third level nonlinear group-by-timepoint PEB (Fig. 3A). In terms of connectivity common to all groups, there was increased indirect nonlinear modulation of AI self-connection, as gated by ACC (parameter 1, Pp >99.9%) in addition to the already identified increased direct nonlinear modulation of VC to AI, as gated by ACC (parameter 3, Pp >99.9%). In terms of group differences in connectivity, EP group showed greater direct nonlinear modulation of VC to AI, as gated by SPC (parameter 24, Pp >99.9%), in addition to the already identified HC group showing greater direct nonlinear modulation of VC to AI, as gated by ACC (parameter 21, Pp >99.9%). In terms of differences explained by timepoint, there was increased modulation of indirect nonlinear modulation of SPC to AI, as gated ACC (parameter 32, Pp >99.9%) and decreased direct nonlinear modulation of VC to AI, as gated by SPC (parameter 36, Pp >99.9%) with timepoint. * Indicates effects where the 90% credible interval does not reach or cross zero.

**Fig. S5**

**A)**


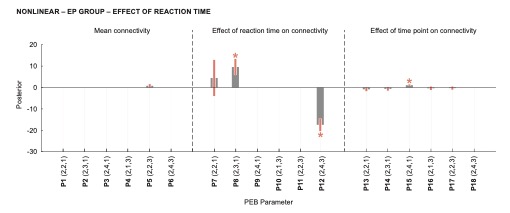


**B)**


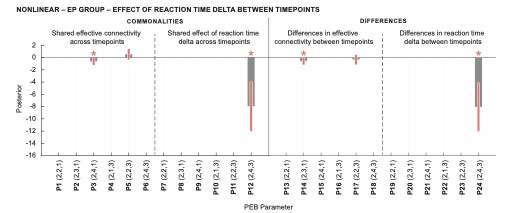


**A** The within EP group second level PEB for timepoint already parsimoniously modelled the effect of timepoint. When effect of timepoint was modelled as the second covariate in the within EP group second level PEB for reaction time there was an increase in the direct nonlinear modulation of VC to AI, as gated by the ACC (Parameter 15, Pp >99.9%). This replicated the result from the within EP group second level for timepoint. **B** Since the second level PEB in Fig. S1A can only model the main effect of reaction time delta, we modelled the interaction of reaction time delta and timepoint with a third level PEB. Consistent with the differences in effective connectivity between timepoints within EP group (Fig. 4A) and the main effect of reaction time delta (Fig., 4B), there was a decrease between timepoints in indirect nonlinear modulation of SPC to AI, as gated by ACC (Parameter 14, Pp 85.4%). Furthermore, reductions in reaction time delta were associated with direct nonlinear modulation of VC to AI, as gated by SPC (parameter 12, Pp 98.7%). * indicates effects where the 90% credible interval does not reach or cross zero.

**Fig. S6**


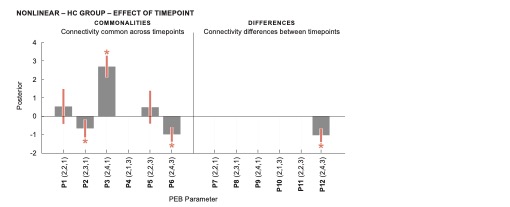


Within HC group shared effects between timepoints included indirect nonlinear modulation of SPC to AI, as gated by ACC (parameter 2, Pp 93%), increased direct nonlinear modulation of VC to AI, as gated by ACC (parameter 3, Pp >99.9%) and decreased direct nonlinear modulation of VC to AI, as gated by SPC (parameter 6, Pp 99%). Within group differences between timepoints included decreased direct modulation of VC to AI, as gated by SPC (parameter 12, Pp >99.9%). It is notable that the within HC group shared effects between timepoints (Fig. S6) mirrored the within EP group differences between timepoint (Fig. 4A), with regards to decreased indirect nonlinear modulation of SPC to AI, as gated by ACC (parameter 2 for HC, parameter 8 for EP) and increased modulation of VC to AI, as gated by ACC (parameter 3 for HC, parameter 9 for EP). Despite an absence of group-by-timepoint interaction, this suggests that the EP group normalized to the HC model in processing stimulus conflict at follow-up. The decreased direct nonlinear modulation of VC to AI, as gated SPC (parameters 6 and 12) was unique to HC group and still suggests a different model for processing of stimuli conflict compared to EP group. Even though there is a decrease in the direct nonlinear modulation of the AI, as gated by SPC, (Parameter 12, Pp >99,9%) for the HC group there were no between group differences in this parameter and for both groups this modulation determined faster reaction times. * indicates effects where the 90% credible interval does not reach or cross zero.

**Fig. S7**

**
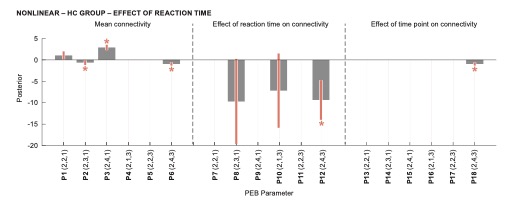
**

Like the second level nonlinear PEB within EP group effect of timepoint, the HC group showed shared effects of indirect nonlinear modulation of SPC to AI, as gated by ACC (parameter 2, Pp 99%), increased direct nonlinear modulation of VC to AI, as gated by ACC (parameter 3, Pp >99.9%) and decreased direct nonlinear modulation of VC to AI, as gated by SPC (parameter 6, Pp >99.9%). Differences explained by reaction time included negative direct nonlinear modulation of VC to AI, as gated by SPC (parameter 12, Pp 99%). Differences explained by timepoint (which were already parsimoniously modelled in the within HC group second level PEB for effect of timepoint) included decreased modulation of direct nonlinear modulation of VC to AI, as gated by SPC (parameter 18, Pp >99.9%). * indicates effects where the 90% credible interval does not reach or cross zero.

**Fig. S8**

**
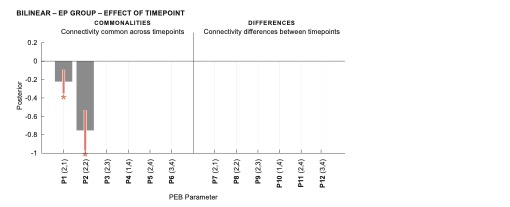
**

Shared effects within EP group of timepoint included decreased linear modulation of ACC to AI (parameter 1, Pp 96%) and decreased linear modulation of the AI self-connection (parameter 2, Pp >99.9%). There were no differences between timepoints. These effects were not commented on in the manuscript due to an absence of main or interaction effects in the third level PEB for effect of timepoint. * indicates effects where the 90% credible interval does not reach or cross zero.

**Fig. S9**


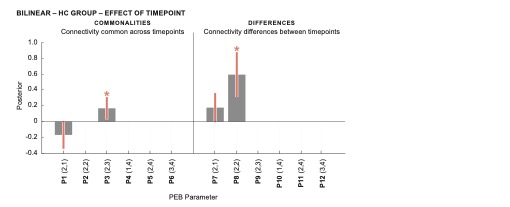


There was a within HC group shared effect of increased linear modulation of SPC to AI (parameter 3, Pp 86%). Differences in connectivity between timepoints included increased linear modulation of the AI self-connection (parameter 8, Pp 99%) from baseline to follow-up. These were not commented on in the manuscript due to absence of main or interaction effects in the third level PEB for effect of timepoint. * indicates effects where the 90% credible interval does not reach or cross zero.

**Fig. S10**

**
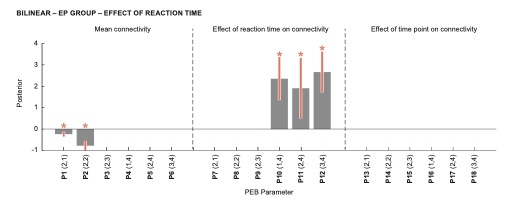
**

Within EP group shared effects for reaction time included decreased linear modulation of ACC to AI (parameter 1, Pp 97%) and decreased linear modulation of the AI self-connection (parameter 2, Pp >99.9%). This replicated the results from the second level PEB within EP group for effect of timepoint. In terms of within group differences for reaction time, there was positive linear modulation of VC to ACC (parameter 10, Pp >99.9%), VC to AI (parameter 11, Pp 92%) and VC to SPC (parameter 12, Pp >99.9%). This was a similar finding to our baseline study (20) where the EP group had reaction time dependency on bilinear modulations in VC to ACC and VC to SPC. There were no differences in linear modulations determined by timepoint. These effects were not commented on in the manuscript due to absence of main or interaction effects in the third level PEB for reaction time. * indicates effects where the 90% credible interval does not reach or cross zero.

**Fig. S11**

**
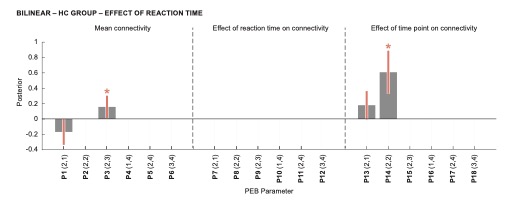
**

There was a within HC group shared effect of increased linear modulation of SPC to AI (parameter 3, Pp 86%). Unlike the EP group, reaction time did not explain any within group differences for the HC group, which was the same finding from our baseline study (20). Like the within HC group effect of timepoint PEB, there was an increased modulation of AI self-connection from baseline to follow-up (parameter 14, Pp 99%). These effects were not commented on in the manuscript due to an absence of main or interaction effects in the third level PEB for effect of reaction time. * Indicates effects where the 90% credible interval does not reach or cross zero.

**References**

1. Castle D, Jablensky A, McGrath J, Carr V, Morgan V, Waterreus A, et al. The diagnostic interview for psychoses (DIP): development, reliability and applications. Psychological medicine. 2006;36(1):69-80.

2. Bush G, Shin LM. The Multi-Source Interference Task: an fMRI task that reliably activates the cingulo-frontal-parietal cognitive/attention network. Nature protocols. 2006;1(1):308-13.

3. Eriksen BA, Eriksen CW. Effects of noise letters upon the identification of a target letter in a nonsearch task. Perception & psychophysics. 1974;16(1):143-9.

4. Esteban O, Markiewicz CJ, Blair RW, Moodie CA, Isik AI, Erramuzpe A, et al. fMRIPrep: a robust preprocessing pipeline for functional MRI. Nature methods. 2019;16(1):111-6.

5. Gorgolewski K, Burns CD, Madison C, Clark D, Halchenko YO, Waskom ML, et al. Nipype: a flexible, lightweight and extensible neuroimaging data processing framework in python. Frontiers in neuroinformatics. 2011;5:13.

6. Tustison NJ, Avants BB, Cook PA, Zheng Y, Egan A, Yushkevich PA, et al. N4ITK: improved N3 bias correction. IEEE transactions on medical imaging. 2010;29(6):1310-20.

7. Avants B, Anderson C, Grossman M, Gee J. Symmetric normalization for patient-specific tracking of longitudinal change in frontotemporal dementia. Med Image Anal. 2008;12:26-41.

8. Zhang Y, Brady M, Smith S. Segmentation of brain MR images through a hidden Markov random field model and the expectation-maximization algorithm. IEEE transactions on medical imaging. 2001;20(1):45-57.

9. Fischl B, Sereno MI, Dale AM. Cortical surface-based analysis: II: inflation, flattening, and a surface-based coordinate system. Neuroimage. 1999;9(2):195-207.

10. Klein A, Ghosh SS, Bao FS, Giard J, Häme Y, Stavsky E, et al. Mindboggling morphometry of human brains. PLoS computational biology. 2017;13(2):e1005350.

11. Fonov V, Evans A, Mckinstry R, Almli C, Collins D. Unbiased nonlinear average age-appropriate brain templates from birth to adulthood. Neuroimage 47 (Suppl. 1), S102. 2009.

12. Cox RW. AFNI: software for analysis and visualization of functional magnetic resonance neuroimages. Computers and Biomedical research. 1996;29(3):162-73.

13. Greve DN, Fischl B. Accurate and robust brain image alignment using boundary-based registration. Neuroimage. 2009;48(1):63-72.

14. Jenkinson M. Bannister P, Brady M, and Smith S. Improved optimization for the robust and accurate linear registration and motion correction of brain images Neuroimage. 2002;17:825-41.

15. Power JD, Mitra A, Laumann TO, Snyder AZ, Schlaggar BL, Petersen SE. Methods to detect, characterize, and remove motion artifact in resting state fMRI. Neuroimage. 2014;84:320-41.

16. Behzadi Y, Restom K, Liau J, Liu TT. A component based noise correction method (CompCor) for BOLD and perfusion based fMRI. Neuroimage. 2007;37(1):90-101.

17. Satterthwaite TD, Elliott MA, Gerraty RT, Ruparel K, Loughead J, Calkins ME, et al. An improved framework for confound regression and filtering for control of motion artifact in the preprocessing of resting-state functional connectivity data. Neuroimage. 2013;64:240-56.

18. Lanczos C. A precision approximation of the gamma function. Journal of the Society for Industrial and Applied Mathematics, Series B: Numerical Analysis. 1964;1(1):86-96.

19. Abraham A, Pedregosa F, Eickenberg M, Gervais P, Mueller A, Kossaifi J, et al. Machine learning for neuroimaging with scikit-learn. Frontiers in neuroinformatics. 2014;8:14.

20. Burgher B, Whybird G, Koussis N, Scott JG, Cocchi L, Breakspear M. Sub-optimal modulation of gain by the cognitive control system in young adults with early psychosis. Translational psychiatry. 2021;11(1):1-9.
